# Routing in triple loop circulants: A case of networks-on-chip

**DOI:** 10.1016/j.heliyon.2020.e04427

**Published:** 2020-07-17

**Authors:** Aleksandr Yu. Romanov, Vladimir A. Starykh

**Affiliations:** National Research University Higher School of Economics, 34 Tallinskaya Ulitsa, Moscow, 123458, Russian Federation

**Keywords:** Electrical engineering, Topology, Computer architecture, Algorithm design, Very-large-scale integration, Computer-aided engineering, Network-on-chip, Dijkstra's algorithm, Triple loop circulant, Routing algorithm.

## Abstract

In this paper we propose and analyze various approaches to organizing routing in a triple loop circulant topologies as applied to networks-on-chip: static routing based on universal graph search algorithms, such as Dijkstra's algorithm and a possible implementation using Table routing; algorithms created analytically based on an engineering approach with taking into account the structural features of triple loop circulant graphs (Advanced clockwise, Direction selection); an algorithm created on the basis of a mathematical analysis of graph structure and solving the problem of enumerating coefficients at generators (Coefficients finding algorithm). Efficiency, maximum graph paths, occupied memory resources, and calculation time of the algorithms developed are estimated. Comparison of various variants of the algorithms is made and recommendations on their application for the development of networks-on-chip with triple loop circulant topologies are given.

It is shown that Advanced clockwise and Direction selection algorithms guarantee that the packet reaches the destination node, but often in more steps than the shortest path. Nevertheless, they themselves are simpler and require less hardware resources than other algorithms. In turn, Coefficients finding algorithm has great computational complexity, but is optimal and, in comparison with Dijkstra's algorithm, is much simpler for RTL implementation which reduces network-on-chip routers resources cost.

## 1 Introduction

Development of elemental base of electronics and communication technologies opens up new opportunities and poses new challenges for their use to achieve new levels of computing system performance [1, 2, 3]. One of the fundamental problems in the field of Multiprocessor Systems-on-Chip (MPSoCs) [4, 5] at present is the construction of communication structures and algorithms for exchanging data in Networks-on-Chip (NoCs) [6] of new generations. Development of communication tools allows combining a large number of processors into compact (dense) structures with a minimum diameter, minimum exchange delays, maximum bandwidth capability, reliability, and survivability with low hardware costs and power consumption within a single chip. Within the framework of this problem, it is required to develop and study new optimal communication structures and interaction algorithms for NoCs.

New classes and families of such optimal structures are constructed on the basis of both regular [7, 8, 9] and irregular [10] network topologies. The NoC topology itself has a decisive influence on NoC performance [11, 12]. Therefore, the search for new topologies is acute, and circulant topologies look promising [13] since they have better characteristics compared to classical topologies [14]. At the same time, standard routing approaches have a rather low efficiency. So, using the classic Dijkstra's algorithm for routing in NoCs is too resource-intensive due to the complexity of implementing the algorithm at the level of a NoC router or IP core [15, 16]. In case of Table routing, it is necessary to store all the routing information at the level of each router, and this is also a resource-intensive solution [17].

For the proposed classes of structures, it is required to study the organization of interactions and develop effective communication algorithms for point-to-point and multiple exchanges. The objective of this work is to develop simple algorithms of various types which can be implemented as RTL state machines at the router level in NoCs.

## 2 Background

The analysis of two-dimensional circulants as a kind of topologies for NoC development, given in previous works, show that ring circulants are highly competitive in their characteristics with optimal circulants of the same order [18]. Moreover, in general case, due to their simpler structure, they allow the use of simpler and more efficient routing algorithms. And only for the family of optimal circulants of type $C\left(N;\;D,\;D+1\right);\;D=\sqrt{N/2}-1,N>2$ [14, 19], having a number of unique properties, it was possible to offer a better routing algorithm compared with the one for ring circulants.

In case of three-dimensional circulant, there is no such graph families that could be described using a formula. Their synthesis is carried out using specialized software [20] as a result of which a volume dataset of optimal graphs is obtained [21]. At the same time, a routing algorithm that would perform well for graphs with any number of nodes could not be proposed. Moreover, as in the case of two-dimensional circulants [18], ring circulants characteristics basically coincide and differ insignificantly in average distance between nodes and diameter only at some graph orders [21]. Thus, for some tasks, where it is required to simplify the routing algorithm, it is justified to use ring circulants because they provide a significant gain in their characteristics in comparison with the classical regular 3D-mesh and 3D-torus topologies [19].

### 2.1 Triple loop circulants

**Figure 1:**
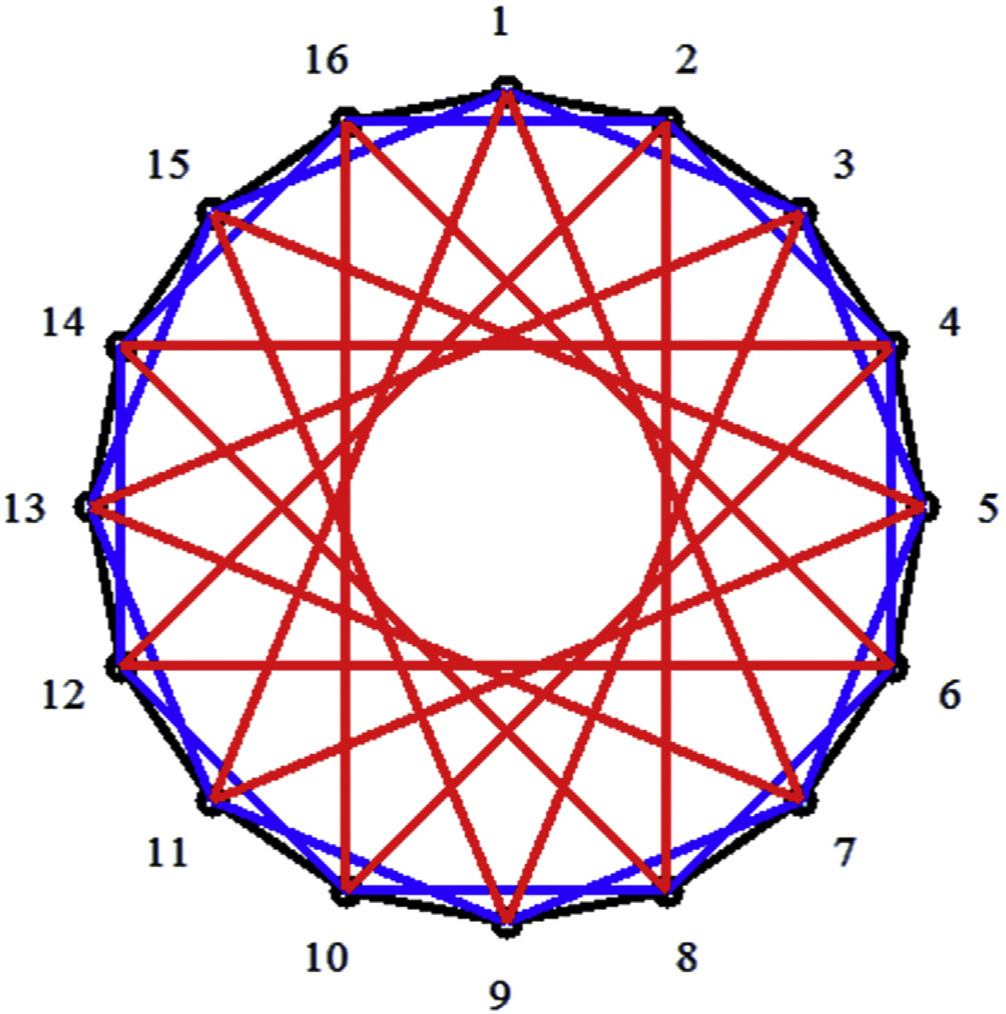
Ring circulant C(16; 1,  2,  6).

Circulant graphs of type $C\left(N;\;1,\;s_{2},\;s_{3}\right),$ where $2\;\leq \;s_{2}\;<\;s_{3}\;<N$, are called triple loop circulants which are a special case of ring graphs [22]. An example of such a graph is shown in Figure 1.

Three-dimensional circulants are a promising topology for NoC design. As for the 3D-mesh and 3D-torus topologies [14], routers in such networks contain 6 external ports, but the presentation of such topologies is possible in two-dimensional form which is best suited for modern ASICs and FPGAs performed using planar technology. In addition, circulants have better diameter and average distance between nodes compared to classical regular topologies [18].

## 3 Study area

### 3.1 Static routing in NoCs with triple loop circulant topologies

Most NoCs use pair routing [16], when a packet is sent from a data source router to a destination node router. To organize such routing, one can use any algorithm for finding the shortest path, for example, Dijkstra's algorithm [20]. Usually a static type of routing is used [17], where each router of each node stores a list every element of which is one of the network nodes and represents a different list with the numbers of the nodes to which this node is connected. In addition, each router knows its own serial number. The input to the router, which will have to transmit the data packet, receives the number of the destination node (receiver router). The router knows the network structure and, therefore, can calculate the shortest path using one of the algorithms.

The essence of Dijkstra's algorithm [16] is as follows: each vertex is associated with a label that contains the minimum known distance from this vertex to vertex A (if the distance is unknown, then it is considered equal to infinity or a sufficiently large number so that it can be considered infinitely large). The algorithm step by step iterates over each vertex and checks whether it is possible to reduce the distance (label) from the starting node to the neighbor vertex using this vertex (the path from the starting vertex to the current one). Dijkstra's algorithm performs until all the vertices are visited.

Dijkstra's algorithm is quite universal and suitable for any graphs on the basis of which circulants are developed, and, therefore, it is suitable for any types of circulants including ring ones with any number and value of generators [15]. The main problem of this algorithm is that with an increase in the number of nodes, the runtime and memory consumption increase significantly. Therefore, there is a need to develop a specialized algorithm that would allow routers to calculate the next packet step on the network based on its routing information. There are few works in the literature devoted to the search for the shortest paths in three-dimensional circulants. There are solutions [23, 24] for particular circulant families of order $N=\text{O}\left(3d^{2}\right)$, where d – diameter, and work [22] presents a simple analytical method for finding the shortest path in circulants of maximum order for a given diameter. There is neither universal routing algorithm for three-dimensional circulants nor for the subfamily of ring circulants.

## 4 Design

### 4.1 Development of specialized routing algorithms for NoCs with triple loop circulant topologies

The number of router ports is defined by the degree of vertices of the graph as p=2k, where k – graph dimension (generators count) [14]. So, the router of circulant of type $C\left(N;\;1,\;s_{2},\;s_{3}\right)$ has 6 interconnections with other routers.

The most obvious algorithm for navigating in circulant networks is the Table routing algorithm described in [20]. The routing table is a square matrix NxN, where N – number of nodes (routers); the cells contain ports numbers to which the packet must be sent so that it reaches the destination node. Each router stores only its own row from the table. According to work [18], the amount of memory occupied by routing table is:

(1) $$M=N^{2}\cdot \log_{2}p,$$

where N – number of nodes; $\log_{2}p$ – memory in bits to store the indexes of ports of the router; p – vertex degree (router port count).

On the one hand, the use of a table routing algorithm requires the storage of large amounts of data; on the other hand, the implementation of such an algorithm as RTL state machine does not take up much space on the chip and is quite simple.

#### 4.1.1 Clockwise algorithm

For routing in triple loop circulant networks, we propose an algorithm based on an iterative calculation of the route between the nodes in which each router makes a decision about switching a packet to the next router only by one step. Since the circulants are symmetric, for any node, its sequence number is not important; it is the distance (in hops) to other nodes that matters. Therefore, to reduce the size of address (distance) field, in the packet, the difference between the node numbers (data source and receiver) is transmitted as the address. The load on the packet (size of address field, bit) can be calculated by the following formula:

(2) $$P=\log_{2}N$$

The router also stores N, $s_{2}$ and $s_{3}$; so, the total size of the stored data is:

(3) $$M=N\text{∗}\left(\log_{2}N+\log_{2}\frac{N}{2}+\log_{2}\left(\frac{N}{2}-1\right)\right),$$

where $\log_{2}N$ – memory in bits to store N; $\log_{2}\frac{N}{2}$ and $\log_{2}\left(\frac{N}{2}-1\right)$ – memory in bits to store generators $s_{2},\;s_{3}$ because generator $s_{3}$ will surely be less than N [4], and generator $s_{2}$ will be at least by one less than $s_{3}$.

By analogy with [18], the transition is calculated as follows: the direction clockwise or counterclockwise of transition is calculated, and then one of the generators is selected. The clockwise motion is chosen if the difference between the numbers of source and receiver nodes is less than half of N. Otherwise, the opposite direction is chosen. When a clockwise motion, the procedure of generator choice is as follows:

− the transition will be by the larger generator while the difference between the source and receiver nodes is greater than value $s_{3}$;
− if the difference is greater than $s_{2}$, but less than $s_{3}$, the transition will occur over generator $s_{2}$, otherwise – over generator $s_{1}=1$.

At every step of the algorithm, the distance is changed by subtracting the length of the generator over which the transition will be made. The zero-value distance is a criterion that the packet reached its destination. If a counterclockwise motion is chosen, the algorithm for selecting the current step is the same, but the generators and the difference between the value of the number of nodes in the network and the distance value are compared. Before the transition, the length of the generator over which the transition will occur is added to the distance field in the head flit. The criterion for the end of the packet transmission (in this case) is the equality of the address field of the head flit to the number of nodes in the network. Proposed algorithm has much in common with a similar algorithm for two-dimensional ring circulants described in [18]. Its description is given below:

**algorithm Find_Route_Triple_Loop_Circulant_Clockwise** is

**Input:** startNode – start node, endNode – end node, N – count of nodes, $s_{1}$ – first generator, $s_{2}$ – second generator, $s_{3}$ – third generator.

**Output:** startNode – next start node.

1: S←endNode–startNode
2: **If** S=0 **then**
3: **return** startNode
4: **If** S<0 **then**
5: S←S+N
6: **If** $S\leq \frac{N}{2}$ **then**
7: **If** $S\geq s_{3}$ **then**
8: $startNode\leftarrow \left(s_{3}+startNode\right)modN$
9: **else**
10: **If** $S\geq s_{2}$ **then**
11: $startNode\leftarrow \left(s_{2}+startNode\right)modN$
12: **else**
13: $startNode\leftarrow \left(s_{1}+startNode\right)modN$
14: **else**
15: S←N−S
16: **If** $S\geq s_{3}$ **then**
17: $startNode\leftarrow \left(N-s_{3}+startNode\right)modN$
18: **else**
19: **If** $S\geq s_{2}$ **then**
20: $startNode\leftarrow \left(N-s_{2}+startNode\right)modN$
21: **else**
22: $startNode\leftarrow \left(N-s_{1}+startNode\right)modN$
23: **If** startNode=0 **then**
24: startNode←N
**25: return** *startNode*

The presented algorithm is not optimal, because in some cases, it will offer paths whose length (in hops) is greater than the network diameter, but it will significantly save the memory occupied by the router.

It is possible to slightly optimize this algorithm as follows: compare the difference between the source and receiver with $\frac{s_{3}+s_{2}}{2}$ and $\frac{s_{1}+s_{2}}{2}$. If the difference is greater than the first value, the transition will be made over generator $s_{3}$; if it is between these values, the transition will be made over $s_{2}$, otherwise – over $s_{1}.$

Total size of the stored data for this algorithm will be equal to the basic one (3). This algorithm works slightly better than the previous algorithm, but still not efficiently enough.

#### 4.1.2 Direction selection algorithm

Development of the proposed approach is the Direction selection algorithm which changes the direction of motion by analogy with work [18]. The destination node index is stored as an address in the head flit. The load on the packet doesn't change (2). Every router stores its index: N and $s_{2},s_{3}$; so, the resulting formula to calculate the amount of the stored data is:

(4) $$M=N\text{∗}(2\text{∗}\log_{2}N+\log_{2}\frac{N}{2}+\log_{2}\left(\frac{N}{2}-1\right).$$

The work of the algorithm consists of 2 stages. Firstly, the sequence of transmission of numbers of nodes of the packet source and receiver is selected; then they are transferred to the second stage of the algorithm. This is possible due to the fact that the graph is non-oriented, and its vertices are transitive [14]. This trick simplifies the algorithm by working only with positive numbers. Also, on the first stage, the received direction of the packet is normalized, and on the second stage of the algorithm, the next step of motion of the packet is calculated directly. The algorithm proposed has much in common with a similar algorithm for two-dimensional ring circulants described in [18]. Its description is given below:

**algorithm Find_Route_Triple_Loop_Circulant_Direction_Selection** is

**Input:** startNode – start node, endNode – end node, N – count of nodes, $s_{1}$– first generator, $s_{2}$ – second generator, $s_{3}-\text{third generator}$.

**Output:** startNode – next start node.

1: **If** startNode>endNode **then**
2: $startNode\leftarrow startNode-Step\left(endNode,startNode,N,s_{1},s_{2},s_{3}\right)$
3: **else**
4: $startNode\leftarrow startNode+Step\left(startNode,endNode,N,s_{1},s_{2},s_{3}\right)$
5: **If** startNode>N **then**
6: startNode←startNode−N
7: **else**
8: **If** startNode≤0 **then**
9: startNode←startNode+N
10: **return *startNode***

**function Step** is

**Input:** startNode – start node, endNode – end node, N – count of nodes, $s_{1}$ – first generator, $s_{2}$ – second generator, $s_{3}-\text{third generator}$.

**Output:** the function returns the best step (direction is also selected)

1: bestTurnR←0,stepR←0,bestTurnL←0,S←endNode−startNode
2: $R_{1}\leftarrow \frac{S-1}{s_{2}}+S\text{ mod }N,\;R_{2}\leftarrow \frac{S-1}{s_{2}}-S\text{ mod }s_{2}+s_{2}+1$
3: $R_{3}\leftarrow \frac{S-1}{s_{2}}-\left(s_{2}\text{∗}\left(\frac{S}{s_{2}}+1\right)-\text{S}\right)+s_{2}+1,\;R_{4}\leftarrow \frac{S-1}{s_{3}}-S\text{ mod }s_{3}+s_{3}+1$
4: $R_{5}\leftarrow \frac{S-1}{s_{3}}-\left(s_{3}\text{∗}\left(\frac{S}{s_{3}}+1\right)-\text{S}\right)+s_{3}+1$
5: **If** $R_{3}>R_{2}$ **then**
6: $R_{3}>R_{2}$
7: **If** $R_{5}>R_{4}$ **then**
8: $R_{5}>R_{4}$
9: **If** $R_{1}<R_{3}$ **then**
10: **If** $R_{1}<R_{5}$ **then**
11: $bestTurnR\leftarrow R_{1}$, $stepR\leftarrow s_{1}$
12: **else**
13: $bestTurnR\leftarrow R_{5}$, $stepR\leftarrow s_{3}$
14: **else**
15: **If** $R_{3}<R_{5}$ **then**
_16:_ $bestTurnR\leftarrow R_{3}$, $stepR\leftarrow s_{2}$
17: **else**
18: $bestTurnR\leftarrow R_{5}$, $stepR\leftarrow s_{3}$
19: S←endNode−startNode+N
20: $L_{1}\leftarrow \frac{S-1}{s_{2}}+S\text{ mod }N$, $L_{2}\leftarrow \frac{S-1}{s_{2}}-S\text{ mod }s_{2}+s_{2}+1$
21: $L_{3}\leftarrow \frac{S-1}{s_{2}}-\left(s_{2}\text{∗}\left(\frac{S}{s_{2}}+1\right)-\text{S}\right)+s_{2}+1$, $L_{4}\leftarrow \frac{S-1}{s_{3}}-S\text{ mod }s_{3}+s_{3}+1$
22: $L_{5}\leftarrow \frac{S-1}{s_{3}}-\left(s_{3}\text{∗}\left(\frac{S}{s_{3}}+1\right)-\text{S}\right)+s_{3}+1$
23: **If** $L_{3}>L_{2}$ **then**
24: $L_{3}>L_{2}$
25: **If** $L_{5}>L_{4}$ **then**
26: $L_{5}>L_{4}$
27: **If** $L_{1}<L_{3}$ **then**
28: **If** $L_{1}<L_{5}$ **then**
_29:_ $bestTurnL\leftarrow L_{1}$, $stepL\leftarrow -s_{1}$
30: else
31: $bestTurnL\leftarrow L_{5}$, $stepL\leftarrow -s_{3}$
32: **else**
33: **If** $L_{3}<L_{5}$ **then**
_34:_ $bestTurnL\leftarrow L_{3}$, $stepL\leftarrow -s_{2}$
35: **else**
_36:_ $bestTurnL\leftarrow L_{5}$, $stepL\leftarrow -s_{3}$
37: **If** bestTurnR<bestTurnL **then**
38: **return stepR**
39: **else**
40: **return stepL**

The proposed algorithm takes into account cycles and situations when it is more reasonable to first move over the highest generator and then return over the middle one. However, when checking the algorithm on graphs, it has turned out that it is still not always able to guarantee that the packet will move along the shortest path between the nodes.

Therefore, another variant of algorithm (close in the principle of operation to Dijkstra's algorithm) was developed, however, with taking into account the peculiarities of considered circulants.

#### 4.1.3 Algorithm for finding coefficients at graph generators (coefficients finding algorithm)

It is possible to represent finding the shortest path for the topology based on a ring circulant as the following optimization problem:

(5) $$N\text{·}k+s=a_{1}+a_{2}s_{2}+a_{3}s_{3},$$

where k – number of cycle considered (may be negative); s– path length from source node to destination node; $s_{2}$, $s_{3}$ – generators; $a_{1}$, $a_{2}$, $a_{3}$ – coefficients at generators $s_{1}$, $s_{2}$, $s_{3}$ respectively.

This task is similar to the routing algorithm in undirected double loop networks proposed in [25], but for triple loop circulant topologies.

The optimization task is to minimize the sum of absolute values of coefficients $a_{1}$, $a_{2}$, $a_{3}$. Were all the variables expressed in terms of variable $a_{1}$, there would be an equation with the three unknowns $a_{2}$*,* $a_{3}$*,* k remained.

(6) $$a_{1}=a_{2}s_{2}+a_{3}s_{3}-N\text{∗}k-s.$$

Next, it is chosen, in which limits variables $a_{2}$*,* $a_{3}$*, k* will change; then (using simple round robin) value $a_{1}$, is found; after that, the set of coefficients, sum of which is the smallest, is chosen.

Total size of the stored data for such an algorithm is calculated by the formula:

(7) $$M=N\cdot \left(2\log_{2}N+\log_{2}\frac{N}{2}+\log_{2}\left(\frac{N}{2}-1\right)+\log_{2}\alpha +\log_{2}\beta +\log_{2}\tau \right),$$

where α,β,τ – coefficients responsible for the number of cycles and generators that will be considered in the algorithm, respectively; $\log_{2}\alpha +\log_{2}\beta +\log_{2}\tau$ – required amount of memory in bits to store coefficients α,β,τ.

Coefficient τ is set manually as the number of cycles that can be passed in both directions. Then coefficients $\alpha =\left[\frac{\tau \cdot N}{s_{3}}\right]$ and $\beta =\left[\frac{\tau \cdot N}{s_{2}}\right]$.

**function Find_Route_Triple_Loop_Circulant_Coefficients** is

**Input:** startNode – start node, endNode – end node, N – count of nodes, $s_{1}$ – first generator, $s_{2}$ – second generator, $s_{3}-\text{third generator}$.

**Output:** the function returns the best coefficients a_1_, a_2_, a_3_

1: bestA1←maxint,bestA2←maxint,bestA3←maxint
2: S←endNode−startNode,a1←0
3: $zero\leftarrow 10,\;alpha\leftarrow \left(zero*N\right)divs_{3},\;beta\leftarrow \left(zero*N\right)divs_{2}$
4: **For all** $k\in \left(-zero,\;zero\right):$
5: **For all** $a_{3}\in \left(-alpha,\;alpha\right):$
6: **For all** $a_{2}\in \left(-beta,\;beta\right):$
7: $a_{1}\leftarrow k*N+S-a_{3}*s_{3}-a_{2}\text{∗}s_{2}$
8: **If**$\left|a_{1}\right|+\left|a_{2}\right|+\left|a_{3}\right|<\left|bestA1\right|+\left|bestA2\right|+\left|bestA3\right|$ **then**
9: bestA1←a1,bestA2←a2,bestA3←a3
10: **return** bestA1,bestA2,bestA3

## 5 Experimental

### 5.1 Testing of algorithms proposed

To evaluate the performance of the algorithms, it is proposed to use an efficiency criterion that takes into account the number of steps required for a packet to establish broadcast routing. Dijkstra's algorithm is taken as the optimal algorithm [16] which surely finds the shortest path in any connected graph. Therefore, performance criterion is determined by the formula [18]:

(8) $$K=\frac{\sum_{i=1}^{N-1}HD_{\left(0-i\right)}}{\sum_{i=1}^{N-1}HA_{\left(0-i\right)}},$$

where $\sum_{i=1}^{N-1}HA_{\left(0-i\right)}$ – sum of all route length for the algorithm used; $\sum_{i=1}^{N-1}HD_{\left(0-i\right)}$ – sum of all route length calculated by Dijkstra's algorithm.

For testing the algorithms, we chose ring circulants of type $C\left(N;\;1,\;s_{2},\;s_{3}\right)$, where N determined by the formula $N=n^{2},\;\text{where }N\leq 100,\;and\;N=150,\;200,\;300,\;400,\;500$ (n – natural number to compare the results of work of triple loop circulants with torus and mesh topologies). The tested circulants are optimal and have a minimum diameter among ring circulants with the same number of nodes [20]. Test results are given in Table 1.

**Table 1:** Comparison of efficiency of the algorithms developed.

Circulant	Algorithm			
	Clockwise	Advanced clockwise	Direction selection	Coefficients finding
C (9; 1, 2, 4)	1	1	1	1
C (16; 1, 4, 8)	0.818	1	1	1
C (25; 1, 6, 10)	0.742	0.821	0.939	1
C (36; 1, 8, 15)	0.656	0.687	0.955	1
C (49; 1, 10, 23)	0.527	0.685	0.887	1
C (64; 1, 12, 30)	0.481	0.643	0.909	1
С(81; 1, 15, 37)	0.474	0.646	0.959	1
С(100; 1, 17, 40)	0.441	0.588	0.781	1
С(100; 1, 10, 30)	0.689	1	1	1
С(150; 1, 33, 59)	0.329	0.536	0.795	1
С(200; 1, 56, 87)	0.291	0.525	0.804	1
С(300; 1, 74, 138)	0.148	0.279	0.837	1
С(400; 1, 69, 195)	0.195	0.321	0.968	1
С(400; 1, 65, 199)	0.342	0.368	0.988	1
С(500; 1, 34, 200)	0.537	0.947	0.968	1

For Dijkstra's and Table routing algorithms, the efficiency coefficient will always be 1, since the first one is a reference one, and the second one implies the existence of an optimal path. For the clockwise algorithm, the efficiency strongly depends on the $s_{3}$ and the difference between the generators $s_{2}$ and $s_{3}$ – the larger they are, the lower the efficiency of the algorithm is. As for the Coefficients finding algorithm, the efficiency is always 1.

Table 2 presents the results of comparison of the developed routing algorithms on the length of the maximum path in the graph.

**Table 2:** Comparison of maximum graph paths (*hop*) for various routing algorithms.

Circulant	Algorithm			
	Clockwise	Advanced clockwise	Direction selection	Coefficients finding, Dijkstra's algorithm
C (9; 1, 3, 5)	2	2	2	2
C (16; 1, 4, 8)	4	3	3	3
C (25; 1, 6, 10)	5	4	3	3
C (36; 1, 8, 15)	7	5	5	4
C (49; 1, 10, 23)	10	6	5	4
C (64; 1, 12, 30)	12	7	6	4
С(81; 1, 15, 37)	15	9	6	5
С(100; 1, 17, 40)	17	10	9	6
С(100; 1, 10, 30)	11	7	7	7
С(150; 1, 33, 59)	32	17	11	8
С(200; 1, 56, 87)	55	28	15	12
С(300; 1, 74, 138)	73	37	10	8
С(400; 1, 69, 195)	66	36	13	11
С(400; 1, 65, 199)	66	34	23	9
С(500; 1, 34, 200)	37	19	20	18

Thus, for the clockwise algorithm and its improved version, the resulting diameter differs significantly worse than the diameter obtained using Dijkstra's algorithm (for some cases – several times). The direction selection algorithm shows the best quality, but in some cases, the difference between the diameters reaches 14. The Coefficients finding algorithm shows the same result as the reference algorithm.

The operation of the Coefficients finding algorithm was tested on 502 optimal graphs from dataset [21] obtained using the software described in [20]. As a result, it was confirmed that for given coefficients α,β,τ equal 10, 20 and 30 respectively, the efficiency of the algorithm does not fall below 1.

**Figure 2:**
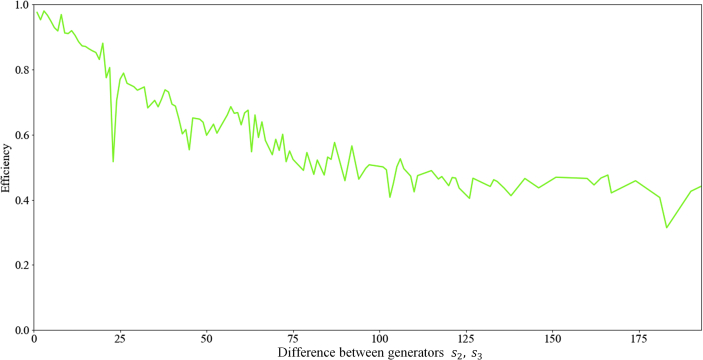
Dependence of the Direction selection algorithm efficiency on difference between generators $s_{2},s_{3}$.

Also, the Direction selection algorithm was tested for the dependence of the efficiency of the algorithm on the difference between $s_{2}\;\text{and }s_{3}$. As a result of testing the algorithm for data [21] among which there were 502 optimal ring circulants, it was found that with an increase in the Difference between generators $s_{2},\;s_{3}$ there is a tendency to decrease the efficiency of the algorithm (Figure 2).

According to the above formulas (3, 4, 7), the calculation of the memory in bits required for the algorithm to work is shown in Table 3.

**Table 3:** Algorithm-occupied memory resources, *bit*.

Circulant	Algorithm			
	Table routing	Advanced clockwise	Direction selection	Coefficients finding
C (9; 1, 3, 5)	243	108	144	270
C (16; 1, 4, 8)	768	192	256	480
C (25; 1, 6, 10)	1875	375	500	850
C (36; 1, 8, 15)	3888	648	864	1368
C (49; 1, 10, 23)	7203	882	1176	1862
C (64; 1, 12, 30)	12288	1152	1536	2432
С(81; 1, 15, 37)	19683	1701	2268	3402
С(100; 1, 17, 40)	30000	2100	2800	4200
С(150; 1, 33, 59)	67500	3600	4800	6900
С(200; 1, 56, 87)	120000	4800	6400	9200
С(300; 1, 74, 138)	270000	8100	10800	15000
С(400; 1, 65, 199)	480000	10800	14400	20000
С(500; 1, 34, 200)	750000	13500	18000	25000

Note: when calculating the memory for the Coefficients finding algorithm of constant α,  β,  τ are chosen equal 10, 20, 30 respectively.

Considered algorithms are implemented in Python 3 which made it possible to estimate the time of their work for finding all the paths in the ring graph. Testing was carried out on a computer with Windows 8.1 operating system, 12 GB RAM, and 2.4 GHz quad-core processor. Table routing algorithm has not been considered, because it does not imply complex calculations. Results of clockwise algorithm are almost the same as those of advanced clockwise algorithm and are, therefore, combined into one column. Obtained indicators of operating time of algorithms in seconds are presented in Table 4.

**Table 4:** Calculation time of the algorithms, s.

Circulant	Algorithm		
	Advanced clockwise	Direction selection	Coefficients finding
C (9; 1, 3, 5)	0.003504	0.006079	28.323078
C (16; 1, 4, 8)	0.003981	0.012421	50.129890
C (25; 1, 6, 10)	0.005578	0.019598	96.979403
C (36; 1, 8, 15)	0.010770	0.036001	116.926932
C (49; 1, 10, 23)	0.018406	0.054717	153.540992
C (64; 1, 12, 30)	0.037407	0.080180	202.219200
С(81; 1, 15, 37)	0.044202	0.113415	261.775398
С(100; 1, 17, 40)	0.090599	0.269699	328.511905
С(150; 1, 33, 59)	0.298190	0.450205	510.957384
С(200; 1, 56, 87)	0.491786	0.728797	656.596207
С(300; 1, 74, 138)	0.867891	0.984096	994.344091
С(400; 1, 65, 199)	0.913595	2.111387	1324.184012
С(500; 1, 34, 200)	0.977516	2.547621	1682.586193

## 6 Future work

Developed algorithms require further research on more circulants in order to determine possible boundary of *N*, when Coefficients finding algorithm ceases to be optimal. RTL synthesis of NoC communication subsystem is also required to confirm the statement obtained in [18] that resource costs calculated using the technique applied in this article correspond to the real amount of ALM blocks and registers consumed on the FPGA. Also, in future, Coefficients finding algorithm as applied to other classes of topologies should be considered, and possibility to formulate (on its basis) a universal approach to routing in any circulants should be analyzed; the latter has not yet been achieved in this work due to complexity of the task and uncertainty about its feasibility in general [14].

Although it is obvious that improving topological characteristics (diameter and average distance between nodes), as well as using adaptive and best propagation routing algorithms improve the functioning of NoCs in general, a thorough study of the impact of proposed routing algorithms on traffic congestion, network latency, throughput, and power consumption is needed. It can be done by using various models of different levels of abstraction and for different traffic profiles.

## 7 Conclusion

The use of triple loop circulant topologies for NoC design is considered. For the triple loop circulants, the following are proposed: a Table routing algorithm, Clockwise algorithm, Direction selection algorithm, and optimal algorithm based on Coefficients finding. It is shown that the classical Table routing algorithm can be replaced by Coefficients finding algorithm at graph generators, since it provides the same number of hops between nodes and is optimal, while its implementation at the hardware level requires significantly less memory resources. Alternatively, with low requirements for NoC throughput, but with limited hardware resources, a clockwise algorithm and its improved version, as well as Direction selection algorithm can be used. They make it possible to reduce the cost of hardware resources by almost 2 times, but lead to a significant increase in the network diameter and average distance between nodes.

A comparison of complexity of algorithms and resources, occupied by synthesized NoC communication subsystems, is made. Since proposed algorithms, unlike classic Dijkstra's algorithm, do not require calculating the entire path of the packet, but determining only the port number for the next step, ensuring that the packet reaches the destination node, they can be easily implemented as RTL state machine in NoC routers.

Despite the fact that the proposed algorithms are applicable to any triple loop circulant topologies, their effectiveness analysis was carried out using optimal circulants. The results obtained allow us to overcome the significant lack of efficient algorithms for routing in triple loop circulants and to expand the application of such topologies on networks-on-chip.

## Declarations

### Author contribution statement

Aleksandr Yu. Romanov: Conceived and designed the experiments; Performed the experiments; Analyzed and interpreted the data; Contributed reagents, materials, analysis tools or data; Wrote the paper.

Vladimir A. Starykh: Analyzed and interpreted the data; Wrote the paper.

### Funding statement

This article is an output of a research project implemented as part of the Basic Research Program at the National Research University Higher School of Economics (HSE University).

### Competing interest statement

The authors declare no conflict of interest.

### Additional information

Data associated with this study has been deposited at GitHub under the accession number https://github.com/RomeoMe5/circulantGraphs.
